# Accurate molecular imaging of small animals taking into account animal models, handling, anaesthesia, quality control and imaging system performance

**DOI:** 10.1186/s40658-015-0135-y

**Published:** 2015-11-11

**Authors:** Christian Vanhove, Jens P. Bankstahl, Stefanie D. Krämer, Eric Visser, Nicola Belcari, Stefaan Vandenberghe

**Affiliations:** Department of Electronics and Information Systems, MEDISIP, Ghent University-iMinds Medical IT-IBiTech, De Pintelaan 185 block B, B-9000 Ghent, Belgium; Department of Nuclear Medicine, Preclinical Molecular Imaging, Hannover Medical School, Carl-Neuberg-Str. 1, 30625 Hannover, Germany; Radiopharmaceutical Sciences/Biopharmacy, ETH Zurich, Institute of Pharmaceutical Sciences, Department of Chemistry and Applied Biosciences, Vladimir-Prelog-Weg 4, CH-8093 Zurich, Switzerland; Department of Radiology and Nuclear Medicine, Radboudumc, 6525 GA Nijmegen, the Netherlands; Department of Physics, University of Pisa and INFN sezione di Pisa, 56127 Pisa, Italy

**Keywords:** Small-animal imaging, Animal models, Animal handling, System performance, Quality control

## Abstract

Small-animal imaging has become an important technique for the development of new radiotracers, drugs and therapies. Many laboratories have now a combination of different small-animal imaging systems, which are being used by biologists, pharmacists, medical doctors and physicists. The aim of this paper is to give an overview of the important factors in the design of a small animal, nuclear medicine and imaging experiment. Different experts summarize one specific aspect important for a good design of a small-animal experiment.

## Background

The European Association of Nuclear Medicine (EANM) symposium entitled Preclinical Imaging: Systems, Acceptance Testing and Implementation and Practical Issues was set up to bring experts from different backgrounds (animal models, handling, quality control and imaging systems) together and summarizes the most important aspects affecting the quality in small-animal imaging experiments with a focus on molecular imaging with PET, SPECT and CT [1, 2]. In this white paper, we summarize in a concise manner the most important aspects of animal imaging for a general scientific public. The final goal of the paper is to communicate for these to researchers a methodology for planning animal experiments and working on a frequent basis in a small-animal imaging facility.

### Justification and focus of paper

**Fig. 1:**
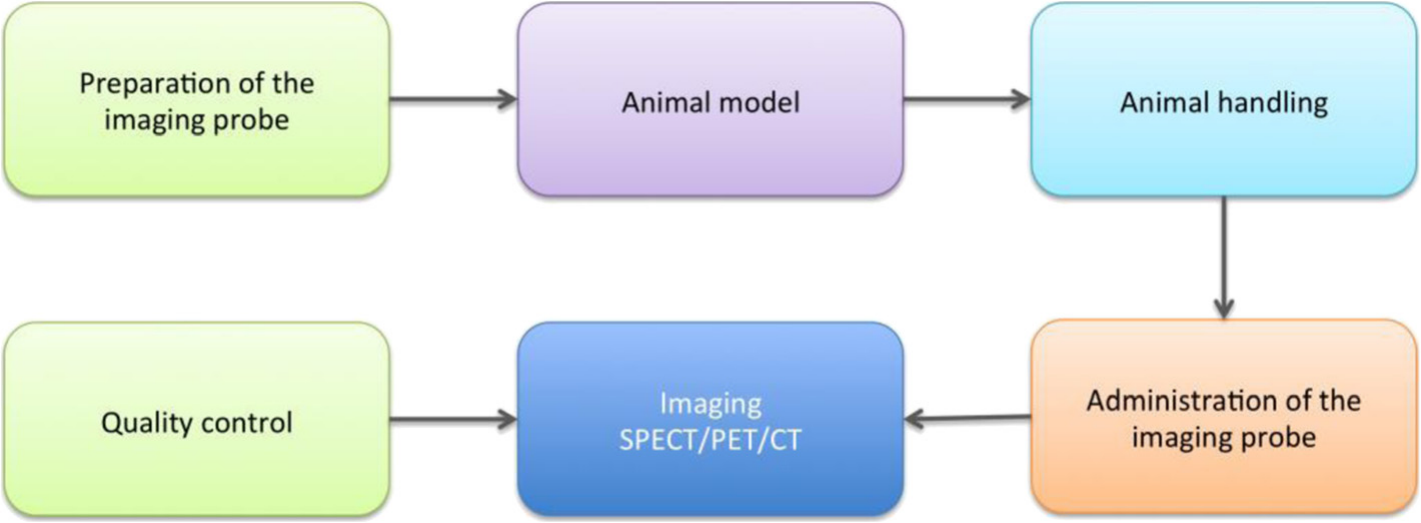
Different steps in animal studies: the preparation of the imaging probe, the selection of the animal model, animal handling and the administration of the imaging probe and imaging and quality control

The final image quality obtained in a molecular animal imaging experiment can be influenced by different factors (Fig. 1): first of all, the isotope, tracer production and labelling are key components. We did not specifically include this here, as it is very similar to human studies. The other main factors are the animal models used, how the animal is treated in between imaging sessions, how it is prepared (and mostly anesthetised before the experiment) right before the imaging study and how it is treated during imaging (heating, monitoring, triggering…). Also, the performance, acquisition and reconstruction settings and quality control of the imaging system have an important effect on the final image. The following sections will describe each of these four aspects, and finally, a concluding paragraph will give some guidelines to the reader. The available animal models and the handling of the animals are described before describing the system performance and quality control.

## Case presentation

### Animal models (J. Bankstahl, Hannover)

Animal models are essential for systemic and functional evaluation of (patho-) physiological processes [3]. Over 75 % of animals used for preclinical research are rodents, which is reflected by the technical specifications of preclinical imaging devices [1, 4, 5]. Today, the spatial resolution of these devices is about 1–1.5 mm. Larger animals, about the size of a cat or bigger, will usually be scanned on clinical imaging systems, which requires special consideration of scanner availability, hygiene and experimental setup.

**Fig. 2:**
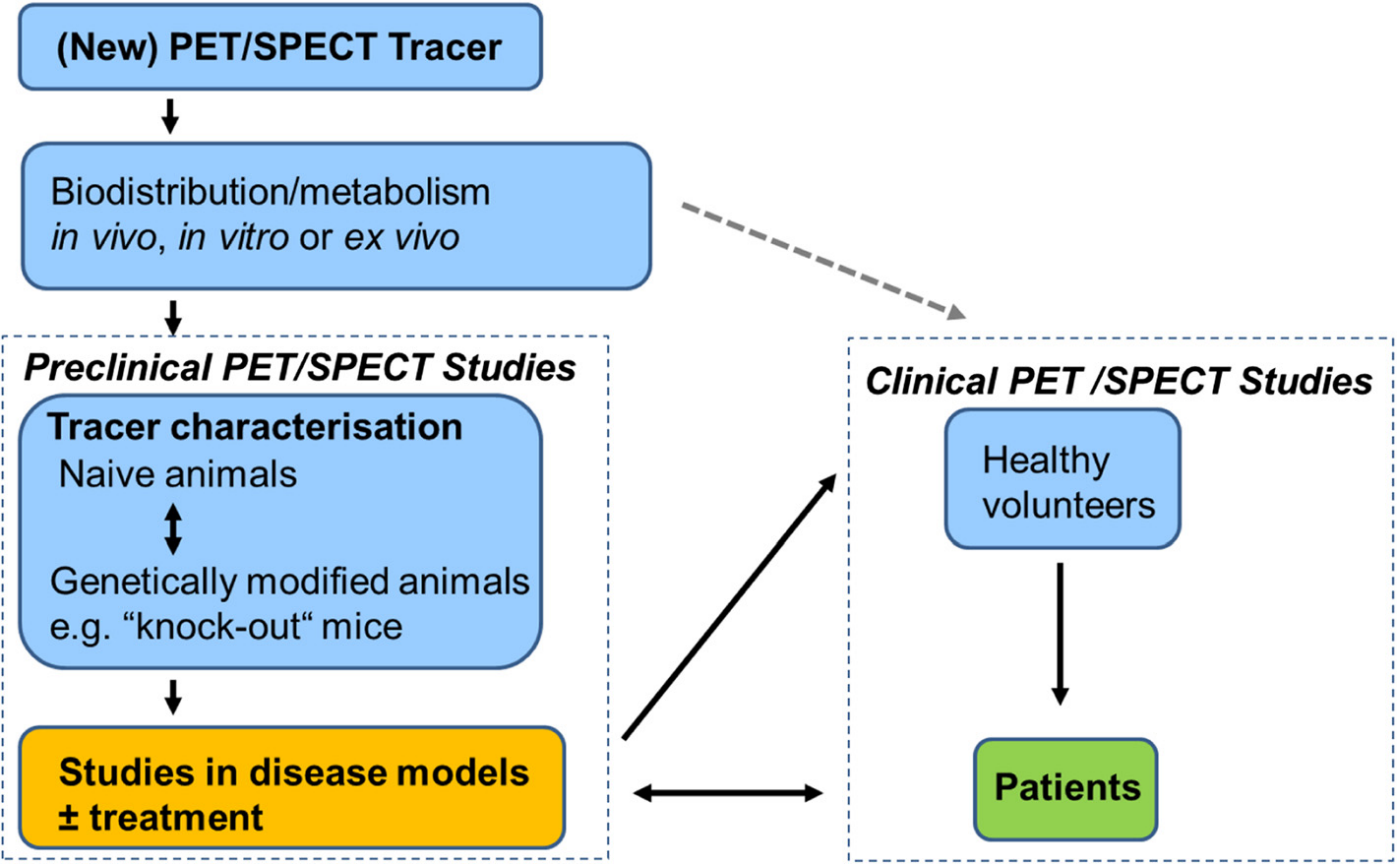
Use of animal models in preclinical radiotracer imaging. The *dotted arrow* indicated the workflow for new tracers before the availability of preclinical scanners

In the early days of radiotracer imaging, due to limited availability of dedicated preclinical imaging systems, animal models were mainly used in *ex vivo* bio-distribution or metabolism studies during the early development of new tracers (Fig. 2). As small-animal cameras have become commonly available, preclinical imaging has become a customary step before performing first-in-man studies. Although animal experiments have distinctly contributed to major advances in life sciences, there are acknowledged limitations with regard to translatability and species differences. Moreover, despite many attempts to improve quality of animal experiments and its reporting, such as the STAIR or the Animal Research: Reporting of In Vivo Experiments (ARRIVE) initiatives [6, 7], it remains advisable to use new tracers in man as early as possible and to utilize animal models in parallel in order to improve protocols and acquire additional knowledge.

As the variety of animal models has vastly increased over the last decades, it is impossible to handle them all single-handed; as such, the imaging specialist will often interact closely with experts in diverse fields of research. The standard approach for evaluation of new imaging agents is to intravenously administer the radiotracer in healthy young adult rodents. One must keep in mind that commercially available rat and mouse strains are inbred to some extent, even if they are described as outbred, and that genetic drift occurs over time even within inbred strains [8, 9]. Consequently, proper control animals are crucial for all experiments, and historic controls comprise large risks for misinterpretation of data. Nevertheless, for baseline evaluation of tracer kinetics or evaluation of interactions with unlabelled compounds, naive animals are the models of choice.

**Fig. 3:**
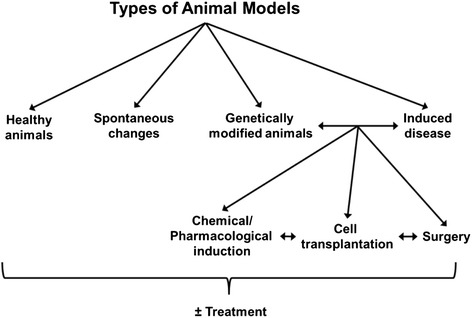
Simplified overview about different types of animal models

In contrast to the situation in patients, and due to the rarity or absence of many spontaneous changes in rodents, most animal models used in preclinical research are induced (Fig. 3). As a general rule, a spontaneous change should be present in at least 20 % of the animals to make preclinical studies feasible. Surprisingly, few preclinical studies deal with the two most obvious spontaneous parameters, i.e. age and gender, whereas both parameters are usually investigated in clinical studies. Other spontaneous changes commonly seen in aged rodents include tumours. Despite some exceptions, such as incidences of >37 % for spontaneous pituitary tumours in male Wistar Hannover rats over one-year old [10], the unpredictable age of disease onset and frequency renders such “natural” models impractical for preclinical research.

Induced disease models include a huge variety of approaches, often employed in combination. Surgical approaches can include vessel occlusion for induction of heart ischemia or stroke, organ removal or transplantation and many more. Chemical disease induction uses various pharmacologically active and/or toxic compounds to selectively kill specific cell types, for instance, beta cells of the pancreas, or to induce a status epilepticus. Cell implantation for disease induction is often used in preclinical oncology. Cell transplantation, especially using different kinds of stem cells, as a treatment option is one of the most exciting areas of current preclinical research [11]. Finally, the wider availability of diverse genetically modified animals gives the opportunity to investigate the biology of gene modification or knockout and gives exciting mechanistic insight into many diseases, which do not spontaneously occur in rodents, such as Alzheimer’s disease [12]. In addition, a gene of an acceptor can be replaced by the gene of a donor, as in the case of transgenic mice, which carry healthy or malfunctioning human genes. All types of genetically modified animals can of course be combined with other induced or spontaneous disease models, as well as multiple treatment approaches, leading to innumerable options for improvement of scientific knowledge.

### Animal handling between, before and during imaging experiments (S. Krämer, Zurich)

#### Legislation, guidelines and experiment planning

Since the year 1959, when Russell and Burch published “The Principles of Humane Experimental Technique” (http://altweb.jhsph.edu/pubs/books/humane_exp/het-toc), ethical aspects and animal welfare have gained much attention in animal research. Russell and Burch introduced the concept of the 3R’s, Replacement, Reduction and Refinement and international, national and institutional legislation, and guidelines nowadays follow these principles, respecting ‘the intrinsic value of an animal (the value in its own right)’. Consequently, research with animals requires justification and a dedicated education and certification. Experiments have to be planned and conducted in accordance with the European (http://eur-lex.europa.eu/legal-content/EN/ALL/?uri=CELEX:32010L0063) or other supranational, national and institutional legislations. Within Europe, the Federation of European Laboratory Animal Science Associations (FELASA) represents its members’ common interests in the furtherance of all aspects of laboratory animal science (http://www.felasa.eu) and when publishing animal experiments, many Journals ask for the statement that experiments were planned, conducted, interpreted and reported according to the ARRIVE guidelines on Animal Research: Reporting In Vivo Experiments (http://www.elsevier.com/__data/promis_misc/622936arrive_guidelines.pdf).

The careful planning of an experiment is the key to keep the number of required animals at a minimum but still reaches a predefined chance to find the true effect of a treatment (see “Number of animals required for an imaging experiment” section). The webpage of the UK National Centre for Replacement Refinement and Reduction of Animals in Research (NC3Rs; http://www.nc3rs.org.uk) provides valuable protocols, not only for power analysis to find the optimal group size for an experiment but also for many other experimental details. Consulting the above and national and institutional webpages and guidelines before planning an experiment with animals is inevitable. In the following, some of the details with relevance for small-animal imaging are discussed.

#### Animal housing

The transport of the laboratory animals to the research facility, the new environment and personnel all cause stress to the animals, which potentially influences the results of an experiment. For this and for reasons of animal welfare, animals should in general be transported to the facility about 1 week before the experiment. Regarding their housing, an enriched environment not only is less stressful to the animals than an unenriched cage but may also give more reproducible results [13]. Enrichment refers mainly to refugees and bedding and nesting material. Bored animals in an unenriched cage often develop stereotypic behaviour. This is repetitive activities at short intervals, and it would come as a surprise if neurotransmitter signalling—the target of most neuroimaging projects—would not be affected in one or the other way in bored animals and animals with stereotypic behaviour. In fact, housing in isolation affects the cognitive function of an animal and is even used as a model of depression in research [14, 15]. According to the animal welfare legislation, the isolated housing of an animal must be avoided if it is not required for a defined reason. However, mixing animals from different cages or any stressful event can result in harmful hierarchy fights, in particular, between males, again, potentially influencing an imaging experiment. The animals should thus be housed in stable groups in enriched cages, taking into account the minimal space requirements for their well-being. The required minimal available space per animal and maximal number of animals per cage are defined in national and European Union (EU) regulations and guidelines. Appendix A (Guidelines for accommodation and care of animals—Article 5 of the convention) to the European Convention for the Protection of Vertebrate Animals used for Experimental and Other Scientific Purposes (ETS No 123) contains tables with information of minimal floor area and cage height besides many more useful information regarding animal housing and animal welfare in general. Using larger cages than the recommended minimal size allows more and diverse enrichment and finally better imitating the natural environment of the animals.

As the housing of male and female animals of the same species or animals from different species in the same room causes or may cause distress to the animals, gender and species need to be separated accordingly [16]. The most common solution is the use of individually ventilated cages that reduce the exchange of odours and dust between cages and racks of cages. Planning a facility, it should also be kept in mind that housing and experimenting need to be separated in two different rooms with a barrier for noise and odours.

Small animals are sensitive to temperature and humidity. Both need to be strictly controlled in an animal facility. Optimal parameters differ depending on the species and strain. The ideal temperature is higher for nude mice than mice with fur. It may be wise to consult the breeder for detailed information on the conditions that the animals are habituated to. In general, rats and mice facilities should have a constant temperature between 20 and 24 °C and a humidity between 35 and 55 % (Appendix A to ETS No 123). Nesting material allows the animals keeping their preferred microclimate. Besides temperature and humidity, ventilation needs strict control to allow sufficient exchange of breathing air, odours and noxious gases and control of local temperature levels.

Mice and rats are active during the dark phase. For practical reasons, their facilities are usually controlled by a light-dark cycle synchronized with daylight, with a 12/12-h interval, with the consequence that the animals are in their inactive phase during experimental time. This should be taken into account in experiments with circadian variations. The light source needs special attention. Even during the light phase, the light needs to be dimmed to prevent degeneration of the sensitive retina of rodents, in particular of albino rodents.

#### Animal handling and education

Animal handling requires a dedicated education, provided by certified national or international organizations. In recent years, FELASA defined categories of educational level and the detailed theoretical and practical contents as well as the respective numbers of hours to be spent on education. This included topics related to ethics, legislation, animal housing, breeding and handling, animal behaviour, health monitoring, evaluation of categories of severity of an experiment, recognition of and methods to reduce pain, distress and other unhealthy conditions, reporting and common experimental techniques. Within the EU, education is now regulated by the directive 2010/63/EU on the protection of animals used for scientific purposes and by the national legislations based on the EU directive. The EU is setting up a platform on education and training in laboratory animal science (Education and Training Platform for Laboratory Animal Science—ETPLAS) with the goals to “help facilitate mutual recognition of laboratory animal science education and training programs, free movement of personnel within the EU and provide stakeholders with information on high quality training programs” (http://www.etplas.eu).

Animal handling can have a significant influence on an experiment as it may cause distress to the animals if it is not properly done. Stress factors include inadequate environmental conditions, inapt contacts between researcher and animals, unfamiliar noise, odours, vibrations and hectic gestures besides the transfer of pathogens between facilities, rooms or cages and the unawareness of signs of animal discomfort, distress or pain. It is thus for the benefit of the animals and of the experiment that the requirement for the respective education is regulated by EU and national law.

#### Hygiene and health monitoring

Imaging platforms can easily become hubs for (potentially) pathogenic microorganisms. Several precautions help to minimize this risk. Animal facilities, and in particular, those transferring animals to others, should monitor the facility according to a list of specified pathogens and keep book in health certificates [17]. Several organizations offer analytical services to test sentinel animals or excreta for contamination with defined microorganisms. Before exchanging animals between facilities, the health certificates need to be exchanged, and only if the receiving facility’s negative list of pathogens is tested negative over a defined time period in the source facility/facilities, the animals can be transferred. Alternatively, and if infrastructure and risk assessment allow, rooms with differing hygiene levels or quarantine rooms can be defined and common instruments cleaned and decontaminated accordingly. However, it should be kept in mind that any infection or its treatment could affect the imaging results.

In addition to the hygiene status, animals need to be monitored for their general well-being. This is usually done according to a pre-defined criteria catalogue and if required individual score sheets, both requiring approval by the respective authorities [18]. Depending on the animal model, score sheets consider, e.g. body weight, appearance of coat, posture, breathing, self-care and social behaviour and appearance of excreta besides disease-related aspects. These score sheets unambiguously define at which state an experiment needs to be terminated and the animal killed. Consulting these score sheets may be of valuable help for the interpretation of unexpected imaging results.

#### Nutrition

Due to their small size, mice have a much higher body surface to body volume ratio than humans resulting in a higher relative water loss through skin. Drinking water should, therefore, not be withdrawn and during longer experiments, lost water needs to be replaced. For PET imaging with the glucose analogue [18 F]FDG, food is often withdrawn for a few hours to reduce the plasma level of glucose, which competes with [18 F]FDG for cell uptake. It depends on the region of interest, e.g. tumour, brain, myocardium, skeletal muscle, to which extent fasting or plasma glucose levels will have an influence on the results [19, 20]. As mice feed frequently and may reach a state of torpor after 7 h of food withdrawal, the fasting period should be kept at a minimum duration [21]. Overnight fasting, what is often seen in publications, is definitely too long if it lasts, e.g. from 5 pm to 10 am, until the experiment is completed. In addition, mice feed most during the dark period, which in most laboratories is overnight.

#### Anaesthesia

Small-animal SPECT or PET scans typically last between 15 and 90 min. In most cases, animals are immobilized during this time by light anaesthesia. Volatile anaesthetics such as isoflurane are preferred as the duration and depth of anaesthesia can easily be controlled, and response to dosage adjustments is immediate. Isoflurane is metabolized to a reactive metabolite that can cause hepatotoxicity, an issue for researchers who are exposed to the anaesthetic on a daily bases. Sevoflurane is a good alternative in this respect, though more expensive. These volatile anaesthetics require a dedicated anaesthesia system including vaporizer and flow metre. They are administered via a carrier gas that is inhaled by the animal. For long anaesthesia periods, the carrier gas may be humidified with water to avoid dehydration of the respiratory airways. Excess and exhaled anaesthetic is aspirated and removed by ventilation or trapped in activated charcoal canisters to reduce immediate and long-term health problems of the experimenters due to isoflurane.

The carrier gas is in most cases a 1:1 mix of oxygen and air that allows regulating the delivery of oxygen to the animal and thus the oxygen pressure in the blood. Hypoxia can be reduced to a minimum or induced with the respective consequences on the experimental outcome. This is of particular interest in hypoxia imaging [22]. Both isoflurane and sevoflurane anaesthesia cause respiratory depression with hypercapnia, acidosis and a decrease in respiration rate within 10 to 50 min after anaesthesia induction [23]. Amongst the frequently used injected anaesthetics are ketamine, often in combination with the muscle relaxant xylazine, furthermore, propofol, chloral hydrate and pentobarbital. Table 1 shows some of their properties [24–26]. The mode of action is not fully understood for most anaesthetics. In particular, those with small, simple structures and high dose interact with several neuroreceptors and other proteins involved in neurotransmitter signalling and in addition affect membrane fluidity.

**Table 1:** Frequently used anaesthetics in small-animal imaging

Anaesthetic	Pharmacology, interaction with	Application route	Typical duration of anaesthesia	Note
Isoflurane, sevoflurane	Various neuroreceptors and other proteins involved in neurotransmission	Inhalation with air/oxygen	Controlled by inhalation	Isoflurane: Hepatotoxicity (staff!)
Ketamine (ketamine/xylazine)	Various neuroreceptors and other proteins involved in neurotransmission	i.p. (rats, mice); i.m. (rats)	20 to 30 min (sleep 1 to 2 h)	Xylazine against muscle rigidity
Propofol	GABA_A_ and various other proteins involved in neurotransmission	i.v. bolus + infusion	Controlled by infusion	
Chloral hydrate, alpha chloralose	Various neuroreceptors and other proteins involved in neurotransmission	i.p.	1 to 2 h (rats)	Alpha chloralose is the acetal between chloral hydrate and glucose
Pentobarbital	GABA_A_ receptor agonist	i.p.	15 to 60 min (mice), 70 to 100 min (rats), (sleep 1 to 3 h)	
Fentanyl/fluanisone–midazolam (hypnorm–dormicum)	Opioid receptor (fentanyl), dopamine receptors (fluanisone), GABA_A_ receptor (midazolam)	i.p.	20 to 70 min (mice), 10 to 110 min (rats), (sleep 1 to 3 h)	
Urethane	Various neuroreceptors and other proteins involved in neurotransmission	i.p.	>24 h	Modest influence on cardiovascular and respiratory systems

For references, see text

*i.p.* intraperitoneal, *i.v.* intravenous

Most anaesthetics not only interfere with neurotransmission in the brain but also affect cardiovascular and respiratory parameters and other physiological functions as discussed above for isoflurane and sevoflurane. Cardiac output, blood pressure and blood flow may change, and due to the high body surface to volume ratio, body temperature may rapidly drop during anaesthesia, requiring heating of the animals. The respiratory rate is reduced and can be used as an indicator of the depth of anaesthesia. Several anaesthetics affect glucose and insulin levels. Anaesthesia-related neurological and peripheral effects can significantly affect the imaging results, and as the effects are often multifactorial, their extent is generally not predictable.

Alstrup and Smith recently reviewed the literature for effects of anaesthesia on PET imaging [27]. Cerebral blood flow is enhanced under isoflurane and ketamine but reduced under propofol anaesthesia. Brain uptake of tracers with high extraction at the blood-brain barrier will be accordingly affected with higher uptake (*K*_1_) at increased and lower uptake (*K*_1_) at reduced cerebral blood flow. As cerebral glucose consumption is typically reduced under anaesthesia, [18 F]FDG uptake in brain is reduced with most anaesthesia protocols, including isoflurane, ketamine, propofol, chloral hydrate and pentobarbital anaesthesia. Several studies report on the effects of anaesthesia on neurotransmitter transporter and receptor imaging. To give two examples, under isoflurane or ketamine/xylazine anaesthesia, accumulation of the dopamine transporter (DAT)-targeting 125I-labelled imaging tracer PE2I was reduced in rat striatum [28] and quantitative PET with the dopamine 1 receptor (D1) targeting [11C]SCH23390 significantly differed between conscious rats and rats anaesthetized with chloral hydrate, ketamine or pentobarbital [29].

As effects of anaesthesia on SPECT and PET results cannot be avoided, it is crucial to standardize protocols as carefully as possible to allow quantitative comparison of the imaging results.

#### Application of tracers and drugs

The small blood volume of a mouse, which is about 58.5 mL per kg body weight (http://www.nc3rs.org.uk) i.e. about 1.5 mL for a 25 g mouse, limits the volume of tracer or drug solution that can be applied. As the total tracer dose needs to be contained in a small volume, relatively high tracer concentrations are required in the formulations for small-animal imaging. Table 2 shows recommended (maximal) volumes applied by various routes for mice and rats according to the NC3Rs guidelines. More detailed information on recommended volumes and sites for substance administration and blood sampling is provided by e.g. K.H. Diehl et al. [30].

**Table 2:** Maximal administration volumes for rats and mice

Route	Rat	Mouse
Intravenous bolus	5 mL/kg (1 mL/200 g rat)	10 mL/kg (0.2 mL/20 g mouse)
Intraperitoneal	10 mL/kg (2 mL/200 g rat)	20 mL/kg (0.4 mL/20 g mouse)
Oral	20 mL/kg (4 mL/200 g rat)	20 mL/kg (0.4 mL/20 g mouse)
Subcutaneous	10 mL/kg (2 mL/200 g rat)	20 mL/kg (0.4 mL/20 g mouse)

Intravenous bolus is the most frequently used administration protocol in small-animal imaging. Larger volumes than maximally tolerated by bolus injection may be applied by intravenous infusion. For kinetic modelling, intravenous infusion over a few minutes may even improve modelling accuracy, as the initial part of the blood curve can be better defined with this protocol. However, the infusion phase should not be too long, as otherwise information on *K*_1_ may get lost. Infusion over about 5 min was optimal for kinetic modelling with [^18^F]FDG in mouse brain.

Besides the volume the pH, osmolality, viscosity and addition of co-solvents are critical factors for substance administration [31, 32]. The recommended pH range for intravenous administration is 2–9. A slow rate of injection in the case of non-physiological conditions reduces the occurrence of adverse effects such as cardiovascular failure. Intraperitoneal administration is more sensitive to non-physiological conditions than intravenous administration where the injected volume is immediately diluted and buffered by the blood pool. For intraperitoneal administration, the pH should be physiological and vehicles limited to saline or water. Tolerated co-solvents for intraperitoneal administration include propylene glycol or polyethylene glycol 400 (PEG400).

#### Blood sampling

Blood sampling during SPECT and PET scans is required for kinetic modelling, to determine blood parameters during the scan, such as the concentration of glucose or the extent of radiotracer metabolism. It is again the small volume of blood that renders blood sampling in small animals particularly challenging. Table 3 shows the maximal volumes of blood that can be withdrawn once or repeatedly. Arterial blood samples may be withdrawn from a femoral or tail artery. For venous blood sampling, several veins are easily accessible. These are, e.g. the tail vein, sublingual vein or the submandibular vein. The tail vein may be cannulated for repeated blood sampling while the sublingual and submandibular veins are better suited for puncture with a sharp needle or lancet [30, 33, 34]. For the recording of an input function for kinetic modelling, protocols with arterio-venous shunts may be preferred as this allows the quantification of radioactivity in blood at a high temporal resolution without blood loss as the blood is guided back into the animal [35].

**Table 3:** Maximal volumes of blood sampling

Species	Body weight	Blood volume per kilogramme body weight	Blood volume at typical body weight	Maximal volume for blood sampling
Human	70 kg	67 mL/kg	4.7 L	
Rat	400 g	64 mL/kg	26 mL	2.6 mL (10 %) at once
				0.26 mL (1 %)/24 h repeatedly
				6.5 mL (25 %) within 28 days
Mouse	25 g	59 mL/kg	1.5 mL	0.15 mL (10 %) at once
				0.015 mL (1 %)/24 h repeatedly
				0.37 mL (25 %) within 28 days

Finally, at the end of each experiment or during a terminal experiment, the animal needs to be euthanized. Methods of “humane killing” are specified by the legislation and the respective guidelines. Common criteria for humane killing are minimal pain, distress and discomfort for the animal and rapid and confirmed death. Bleeding following any other euthanasia method is an unambiguous way to guarantee death of the animal. A common and hardly debated method is decapitation of the anaesthetized animal with a rodent guillotine.

### Number of animals required for an imaging experiment (C. Vanhove, Gent)

With the growing knowledge of new targets related to specific disease and the development of novel molecularly targeted therapies, the number of animals used for non-invasive imaging experiments using SPECT and PET has increased. It is important to note, however, that it is required to take careful attention to the number of animals needed to produce truly useful data from any imaging experiment [36]. The rationale behind choosing a correct sample size is to weigh the benefits; one can gain in information against the cost of increasing the sample size. The number of animals to be acquired to reach statistically significant differences between groups can be computed using a priori statistical power analysis. Software such as InStat (http://www.graphpad.com), G*Power (http://www.gpower.hhu.de) and Interactive Statistical Calculation Pages (http://statpages.org) can be used to simulate the significance of the results. Based on the two-tailed Student’s *t* test with the assumptions of equal standard deviation in the control and treatment/disease groups, and unpaired data analysis if different animals are used for each group, the goal is to calculate the number of animals to achieve a significant difference (*P* < 0.05) between the control and treatment/disease groups [37, 38]. The ability of an in vivo imaging experiment to measure statistically significant differences between a control group and either diseased or treatment groups is further dependent on the intra-animal variability of the acquired data. This variability can be estimated using a test-retest experiment by calculating the ratio between standard deviation and the mean of the two measurements [39]. Table 4 summarizes the number of animals required per group to reach statistical significance in relation to the expected difference between the control and treatment/disease groups and the intra-animal variability of the SPECT/PET data. Given this summary, it can be observed that it is important to decrease the intra-animal variability to a minimum, which can be achieved by standardizing imaging protocols.

**Table 4:** Number of animals required per group to reach statistical significance in relation to the expected difference between control and treatment/disease groups and the variability of the measured data. Data were computed using G*Power by a two-tailed unpaired Student’s *t* test assuming equality of variance in both groups and normal distributions. Furthermore, a false positive rate (type I error) of 5 % was selected, together with a false negative rate (type II error) of 20 %, corresponding to a power of 80 %

Expected change between control and treatment/disease groups in %	Intra-animal variability of the SPECT/PET data in %	Number of animals required per group
20	10	6
20	15	10
20	20	17
25	10	4
25	15	7
25	20	12
30	10	4
30	15	6
30	20	9

### System performance and image reconstruction (C. Vanhove, Gent)

Although SPECT and PET imaging has been used as an important clinical tool for several decades, it can also find a use for preclinical research by imaging small-animal models of human disease [1, 40–44]. Small-animal SPECT and PET systems are often modified versions of their clinical equivalent, where rats and mice form the majority of the experimental animal population. A mouse is approximately 3000 times smaller by volume than a human (25 g versus 75 kg), which means that when clinical systems have volumetric spatial resolutions in the order of 1 cm^3^, preclinical systems require a volumetric spatial resolution of 0.3 mm^3^, or sub-millimetre linear spatial resolution, to visualize sub-compartments of mouse organs.

**Fig. 4:**
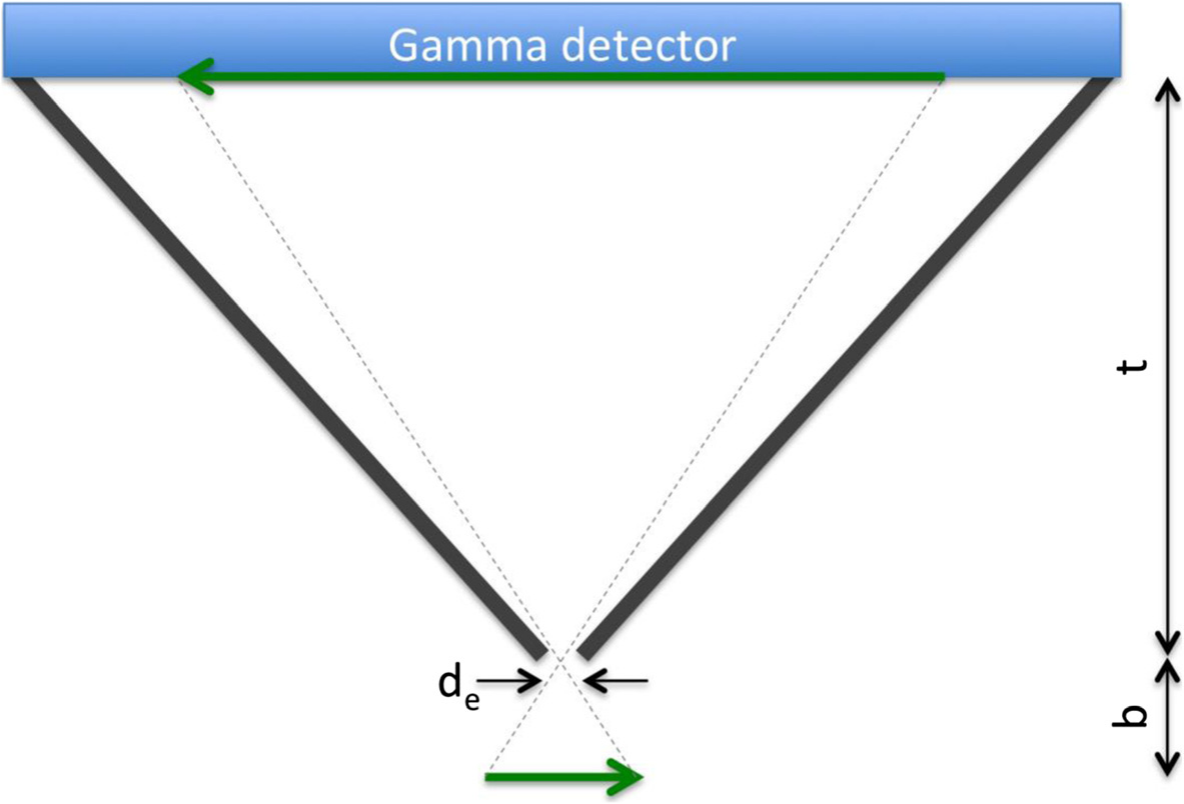
Principles of pinhole collimation with *b* representing the distance between the object and the pinhole opening and *t* representing the distance between the pinhole opening and the gamma detector. *d*_*e*_ denotes the effective diameter of the pinhole opening accounting for penetration of gamma rays through the edges of the pinhole aperture

To obtain this required spatial resolution, most commercial available preclinical SPECT system is based on pinhole collimation. In clinical practice, the pinhole collimator has been mainly used for imaging small volumes, such as the human thyroid. Similar to radionuclide thyroid imaging, the magnification of the projection images due to the pinhole geometry can dramatically reduce information loss as a result of the intrinsic spatial resolution of the gamma detector when an animal is brought in close proximity to the pinhole opening (Fig. 4). The spatial resolution of a pinhole system is approximated by the following formulae:

1 $${\mathit{\mathsf{R}}}_{\mathit{\mathsf{s}}}=\sqrt{{\left[{\mathit{\mathsf{d}}}_{\mathit{\mathsf{e}}}\left(\mathsf{1}+\frac{\mathsf{1}}{\mathit{\mathsf{M}}}\right)\right]}^{\mathsf{2}}+{\left[\frac{{\mathit{\mathsf{R}}}_{\mathit{\mathsf{i}}}}{\mathit{\mathsf{M}}}\right]}^{\mathsf{2}}}$$

Here, *d*_*e*_ is the effective diameter of the pinhole opening taking into account the possible penetration of the gamma rays through the edges of the pinhole aperture and *R*_*i*_ is the intrinsic spatial resolution of the gamma detector. *M* represents the pinhole magnification and is defined as *M* = *t*/*b* (see Fig. 4), with *t* being the distance between the pinhole opening and the detector surface and *b* the distance between the object and the pinhole opening. For most clinical gamma cameras, *R*_*i*_ is typically between 3–4 mm, indicating the magnification larger than 4 might result in sub-millimetre intrinsic spatial resolution. The greater challenge is then to acquire a sufficient number of counts in each individual voxel to support this high spatial resolution and thus to obtain sufficient signal-to-noise ratios comparable to clinical system. The sensitivity of a pinhole system is approximated by:

2 $$\mathit{\mathsf{S}}=\left(\frac{{{\mathit{\mathsf{d}}}_{\mathit{\mathsf{e}}}}^{\mathsf{2}}}{\mathsf{16}{\mathit{\mathsf{b}}}^{\mathsf{2}}}\right)\mathit{\mathsf{N}}$$

where *N* representing the number of gamma detectors used. As a result, the sensitivity of a pinhole SPECT system can be improved by increasing the diameter of the pinhole opening to allow more gamma photons to pass; however, this will be at the cost of a poorer spatial resolution. Further minimizing the distance between the animal and the pinhole will also increase the fraction of detected photons in the field of view, as a result of the increasing solid angle, but it will restrict the size of the field of view. These choices between spatial resolution, sensitivity and size of the field of view need to be carefully examined in view of the species and organs to be imaged. The need for high spatial resolution, in combination with high sensitivity and a realistic size of the field of view has driven the development of multi-detector multi-pinhole systems [45, 46]. These systems increase the sensitivity roughly by factor equal to product of the number of detectors and number of pinholes. The detection efficiency of each individual pinhole in these multi-pinhole systems is lower than that of single-pinhole system, in which entire detector area can be used with high magnification. Though, when carefully designed, the sensitivity can be substantially improved while maintaining the same final spatial resolution and size of the field of view [45]. Another possibility to further increase the sensitivity is to allow overlap between the multiple pinhole projections on the detector surface, which is also referred to as multiplexing [46, 47]. However, it is important to note here that in the overlapping regions, it is impossible to determine through which pinhole a photon travelled before reaching the detector. As a result of this ambiguity, reconstructions of multiplexed multiple-pinhole data converge more slowly than those of non-overlapping projections, resulting in more noisy images. Consequently, the sensitivity advantage of overlapping projections does not necessarily result in an improved signal-to-noise ratio and may only compensate for the introduced ambiguity [47].

Today, all commercial small-animal SPECT systems are based on the multi-detector multi-pinhole geometry using different configurations, including conventional systems where the detector, together with the collimator, rotates around a stationary animal [4, 48], systems where the collimator rotates around a stationary animal and in front of a stationary detector [49] and systems where the animal, collimator and detector are stationary [4, 50].

Preclinical PET systems use similar image formation techniques as human PET systems and can be seen as scaled down versions of clinical systems. Small-animal PET systems also use a cylindrical geometry with a typical diameter of ±15 cm as compared to ±80 cm for clinical PET systems. An advantage of using a smaller detector ring is that it saves detector cost, which allows PET developers to expand the detector rings in the axial direction, improving the geometric detection efficiency of the system. The axial field-of-view of preclinical PET systems is typically ±8 cm, which is more or less equal to the length of a mouse, allowing to measure the whole-body dynamic bio-distribution of a labelled compound in a single scan. To obtain the required spatial resolution for small-animal imaging, scintillation crystals with smaller pixel pitch have to be used. However, fine pixels are difficult to produce and may result in a very expensive imaging system [51]. On the other hand, to provide adequate sensitivity, scintillator crystals should be long. This leads to scintillator arrays made of long and narrow crystals that will increase photon penetration in the scintillator array causing a parallax positioning error that worsens spatial resolution. This parallax error can be mitigated through the use of a few short crystals to replace each single long crystal [52], by modelling the spatially variant detector response functions in the reconstruction algorithm [53] or by using monolithic crystals instead of pixelated detectors. To our knowledge, the best spatial resolution reported for state-of-the-art small-animal PET systems has been about 0.7 mm full-width-at-half-maximum [54] and the highest reported absolute sensitivity at the centre of the field of view is approximately 14 %, which is about six times that of the current state-of-the-art human PET scanners [55].

Next to spatial resolution, two other issues of resolution are also important when dealing with small-animal imaging systems, namely, energy resolution and timing resolution.

The energy resolution is important as better energy resolution improves the discrimination between true unscattered events (i.e. the desirable events for image formation) and Compton scattered events (i.e. undesirable events that affect quantitation and reduce contrast) within the animals being imaged. Because Compton scattering is of decreasing relevance as the photon energy increases, a high-energy resolution is more of a concern in case of SPECT, where lower photon energies are used, compared to PET. Furthermore, SPECT has the capability to simultaneously image multiple radionuclides with different emission energies, and a good energy resolution is here very important to minimize cross talk between the different simultaneously acquired radionuclide measurements. As a result, similar to the clinical, narrower energy windows are used in preclinical SPECT, with a typical width of 10–20 % around the photopeak, while in preclinical PET, very large energy windows are used (350–650 keV).

The timing resolution of a gamma detector is correlated with the decay time and light output of the scintillator. In general, faster decay times corresponded to better timing resolution. Timing resolution is important for separating events in PET and SPECT (count-rate performance) but is of less importance in preclinical imaging because of the lower dose levels applied. However, in preclinical PET, a good timing resolution results in a good coincidence timing that is still important for limiting the collection of random events. Generally, a time coincidence window of approximately 10 ns is used in small-animal PET.

For emission tomography, there are two categories of reconstruction algorithms, namely, analytical and iterative methods. Although, analytical methods are computationally very efficient, they are not suited to deal with the complex multi-detector multi-pinhole SPECT geometry. As a result, iterative reconstruction techniques, such as maximum likelihood expectation maximization, are routinely used in preclinical imaging. Moreover, similar to clinical systems, preclinical PET systems often implement three-dimensional data acquisitions in list mode that are iteratively reconstructed using an MLEM-based algorithm [53]. In addition, the more important need for absolute quantification in preclinical imaging, to better understand the pathophysiology of disease and the impact of treatment, further increases the necessity of iterative reconstruction algorithms. Two criteria characterize the reliability of absolute quantification, namely, accuracy and precision [56]. Iterative reconstruction algorithms can substantially improve both criteria because they include an appropriate statistical model to describe the data, resulting in better noise properties and thus improved precision, and they allow to accurately model image degrading effects such as attenuation, scatter and partial volume effects, resulting in a more accurate representation of the tracer distribution when a sufficient number of iterations are used [57]. Similar to the clinic, partial volume effects can be decreased by modelling the spatially variant detector response [54, 57] and scatter can be estimated by using measurements in additional energy windows [58] or using the more complex single-scatter simulation technique [59]. Attenuation correction requires a co-registered map of attenuation coefficients at the relevant photon energy that for the majority of preclinical systems can be provided by CT [60]. Small-animal CT imaging is usually based on the cone-beam CT geometry [61–63]. In some preclinical SPECT/CT systems, the SPECT and CT components are mounted on the same rotating gantry (in-plane configuration). Another way to combine the two modalities is to keep the two imaging systems separate and to translate the animal between two imaging modalities that are bolted together in a tandem configuration (serial configuration), analogous to clinical SPECT/CT and PET/CT systems. Along with providing an accurate attenuation map, the CT information can be used to provide anatomical localization. In small-animal imaging, this is very important because novel radiopharmaceuticals with high specificity for their target have less non-specific uptake in surrounding organs, suppressing ‘anatomical landmarks’ in the functional images. Moreover, unfamiliar animal anatomies that are not corresponding to human anatomy, and the limited field of view due to the pinhole geometry in SPECT, further necessitate CT imaging.

In summary, the crucial trade-offs in the design of preclinical imaging systems are between spatial resolution, sensitivity and size of the field of view. Other important requirements include accurately co-registered CT images and absolute quantification. It should be emphasized that for accurate quantification, the acquired image data should be corrected for all image-degrading effects and reconstructed with a sufficient number of iterations. It is also important to note that the reconstructed voxel size should be at least smaller than the spatial resolution of the imaging system to minimize partial volume effects due to pixilation.

### Cross-calibration (C. Vanhove, Gent)

In small-animal SPECT and PET imaging experiment, it is a common practice to perform sample counting. Samples can be blood or tissue samples measured using an arterial blood counter or gamma counter, respectively. In both cases, it is important to perform cross-calibrations between SPECT/PET camera, dose calibrator and blood/gamma counter. Cross-calibration is a direct, relative calibration between the institution’s own dose calibrator, SPECT/PET camera and blood/gamma counter. In short, the procedure is as follows: a syringe has to be filled with a radioactive solution with an activity (in Bq) that is close to the injected activity applied during the animal experiment. This syringe should be measured in the institution’s dose calibrator. The solution should then be introduced into a calibration phantom (mostly a cylindrical phantom) with an exact known volume (in mL) filled with water, resulting in a solution with known activity concentration in becquerel/millilitre. Attention should be paid to the homogenization of the solution. A SPECT/PET scan has to be performed of the calibration phantom, and the acquired data should be reconstructed applying the same reconstruction parameters as used during the animal experiment. A volume-of-interest has to be drawn on the reconstructed images in order to determine the average volumetric concentration of activity within the phantom as measured by the SPECT/PET scanner. Samples should then be taken from the calibration phantom, to be measured by the blood/gamma counter. Conversion factors can then be directly derived with which the measurements/counts from the different equipment can be synchronized [64].

### Acceptance testing and quality control (E.P. Visser, Nijmegen)

#### General considerations

Quality control (QC) determines the integrity of nuclear medicine equipment in clinical routine or in (preclinical) research studies. High standards are needed, especially in relation to image quality, quantitative imaging and size or volume measurements in therapy and dosimetry. Also, in pharmacokinetic modelling, used in human or animal research, high standards are needed to guarantee the accuracy of results in terms of pharmacokinetic rate constants.

Although research institutes may have service contracts with their equipment suppliers, several QC procedures should be carried out on a regular basis by the technologists or physicists working in the institute. It should, however, be mentioned that a QC programme can never replace the normal attentiveness of the ‘operator’ in observing equipment problems that are immediately obvious. Instead, a QC programme aims at detecting those changes that happen so slowly that they are normally not detected in everyday use.

#### Selection of tests and criteria to be met

Equipment suppliers generally have many protocols available to assess whether their equipment meets all specifications. Most of these protocols are complex and time-consuming and often need special test equipment and phantoms. To guarantee the normal day-to-day functioning of the equipment, fewer and simpler tests can be used.

A QC programme should provide concrete test results that should be compared with predefined values, usually the ones obtained during acceptance tests. Action threshold values should be defined, as well as a protocol for the actions to be taken whenever certain thresholds are exceeded.

The ‘costs’ and ‘benefits’ of a QC programme have to be balanced. Examples of costs are the downtime of the equipment, personnel costs, costs of phantoms, radioactive sources and the radiation burden to the personnel. The level of benefit is related to the chance that if any degradation has actually occurred, it will be detected by the test and that the consequences of the degradation (for example, a faulty diagnosis, the need to repeat the scan…) can be prevented.

#### Frequency of tests: cost-benefit aspects

In a straightforward approach, fixed test frequencies can be used. However, one risk that too few tests are performed is leading to a greater chance that the equipment may not always be at its optimum condition. On the other hand, in case of too many tests, the equipment is not available for animal research for long periods of time, thus increasing the effective cost of the equipment. For this reason, adaptive frequencies should be used, that is, the test frequencies should be adapted to the reliability of the equipment and the conditions of use.

As a rule of thumb, one should start with a relatively high frequency, which can be halved if no deviations exceeding the action threshold occur during four consecutive tests. This will generally lead to the optimal test frequency. In case of a sudden, unexpected deviation, the frequency should be increased again. However, a certain minimum frequency should still be used; mostly, this coincides with the frequency of (preventative) maintenance of the equipment.

Since small-animal imaging equipment is rather diverse, it is not possible to exactly describe the necessary tests and their recommended frequencies in the present guidelines. Instead, we refer to the recommendations as given by the equipment manufacturers

However, in case of a small-animal study where quantification is (all-)important, special attention must be paid to all checks, and if necessary, adjustments, which are directly related to quantification of activity concentrations in the images. These include the calibration factor (as determined using the same settings as in the small-animal experiment), normalization procedures, detector setup and adjustment of energy peak positions, etc. In these cases, it is advised to perform these tests before each experiment. Finally, it is recommended to perform all available tests after each (major) hard or software upgrade.

#### Action levels

Whereas test procedures and equipment specifications are generally described in a very exact way (e.g. in NEMA procedures), this does not hold for action threshold levels. In general, it is not possible to define absolute values for action levels from ‘first principles’. Instead, action levels are determined by experience with the cost-benefit aspect in mind. With the proper choice of action levels, degradations should not yet have reached a stage where they can be detected in clinical images, but on the other hand, the equipment is not put out of use for readjustments, calibrations, for a too longtime period.

#### NEMA tests

The National Electrical Manufacturers Association (NEMA) has issued four documents dealing with performance measurements of nuclear medicine equipment. These are gamma camera’s (NU1), PET scanners (NU2), non-imaging intra-operative gamma probes (NU3) and small-animal PET scanners (NU4) [5]. It is seen that, unfortunately, NEMA standards do not (yet) exist for small-animal SPECT scanners. The NEMA standards have been created by task forces consisting of members from industry and academia. They are consensus documents, which implies that ‘the information in the publication was considered technically sound by the consensus of persons engaged in the development and approval of the document at the time it was developed’. It is however mentioned in each publication that ‘consensus does not necessarily mean that there is unanimous agreement amongst every person participating in the development of the document’.

Basically, the documents serve to objectively characterize the performance of equipment and to facilitate the comparison of equipment of different vendors. They are not primarily meant for recommendations with regard to QC parameters, procedures and test frequencies but are nevertheless often used as such. In practice, many institutes will select a subset of the NEMA tests to be used for QC, keeping in mind the aforementioned aspects of costs and benefits.

It is interesting to identify as to why small-animal NEMA standards exist for PET and not for SPECT. My personal feeling is that this might be attributed to the much larger variety in parameters with regard to equipment hardware and acquisition and reconstruction settings in small-animal SPECT as compared to PET. These include, for instance, static versus rotating collimators, planar or cylindrical collimators, numbers of pinholes, varying radii of rotation or collimator diameters, using separated or overlapping projections, etc. The variety of photon energy, as opposed to the fixed value of 511 keV in PET, requires different (pinhole) collimator designs. Moreover, attenuation correction in single-photon detection is more of a challenge than in coincidence detection of two collinear photons. All these items could have led to more difficult ‘consensus’ in NEMA standards for small-animal SPECT than for small-animal PET.

#### Guidelines

As stated above, NEMA tests have not primarily been set up as guidelines for acceptance testing and QC. Specifically, the NEMA documents do not specify any recommended test frequency, nor do they present an ‘order of importance’, based on the (pre)clinical relevance of the parameters to be tested. For this purpose, we need guidelines. Several guidelines for acceptance testing and QC of nuclear medicine equipment have already been issued on behalf of the EANM Physics Committee. In both documents, guidelines for small-animal PET are given, but small-animal SPECT is missing.

Table 11 in reference [65] summarizes the acceptance tests for small-animal PET and their purpose and indicates whether a certain test is solely used for acceptance/non-acceptance or whether it also generates reference values to be used in later QC procedures. Several of the tests prescribe to straightforwardly follow the NEMA NU4 procedures. These include sensitivity, count rate performance, image quality, linearity and attenuation and scatter correction.

Table 9 in reference [66] summarizes the routine QC tests for small-animal PET, their purpose and their recommended test frequency, which ranges from daily to yearly. In terms of the cost-benefit trade-off, the daily tests (physical inspection, background count rate, detector check) are of very short duration and do not require expensive tools of phantoms. The weekly tests (energy resolution and pixel identification) at least require a positron source. It will depend on the kind of system/vendor as to which source geometry is described. For the Siemens Inveon, small-animal PET scanner, for example, the pixel identification procedure is part of the ‘detector setup’ and requires a cylindrical phantom. This procedure is somewhat time-consuming, and accordingly, the test frequency could possibly be decreased, based on the procedure of ‘adaptive frequencies’ as described above. The monthly, three monthly and yearly tests all require phantoms or sources and can be relatively time consuming.

#### Absence of guidelines and NEMA standards for small-animal SPECT

NEMA standards and official guidelines for small-animal SPECT are still lacking, which could be related to the considerations as described earlier. Several performance parameters can be easily adapted from PET to SPECT. Parameters that are in common between PET and SPECT are, for instance, image quality, spatial resolution, energy resolution and sensitivity. However, some parameters are ‘PET-only’, such as coincidence timing, and some of them are important for PET but to a much smaller extent for SPECT. These include count rate performance and linearity (related to detector dead time). Since SPECT scanners contain collimators, the rate of photons that reach the detector and photomultipliers is generally much smaller than in PET, and therefore, dead-time and count rate linearity is not an issue for activities normally administered to the animals in SPECT.

On the other hand, the large variety in single-photon emitters with different energies and, accordingly, different collimators requires special attention in SPECT. In principle, this requires that performance parameters be measured for each collimator (and even for each radionuclide) separately. In view of a cost-benefit trade-off, one could however decide to perform QC procedures with a high(er) test frequency for the mostly used collimator and radionuclide and with a lower frequency for other combinations. Furthermore, background radiation is expected to be much more of an issue in SPECT than in PET. Single photons not originating from the animal in the scanner are counted in case the detector is not properly shielded in all directions, which is not the case for coincidence photon detection in PET. Even for multi-pinhole collimators with a large numbers of pinholes, the overall sensitivity in small-animal SPECT is lower than in PET. This results in signal counts (from the animal) that could be close to the background level. A pitfall is the presence of other animals, already injected with a radiopharmaceutical, that are in present in the scanner room and not properly shielded.

#### Fundamental or intrinsic parameters versus image parameters

It could be of importance to distinguish between parameters that can directly be seen or measured in reconstructed images versus parameters that are more ‘fundamental’ to the scanner. The latter type of parameters can also influence image quality, but the way in which this happens is not always directly deducible.

Examples of ‘image parameters’ are spatial resolution as indicated by small structures in the image (small spheres or rods used to calculate recovery coefficients), image uniformity (signal-to-noise ratios, percentage standard deviation), spillover of activity into cold regions and quantification (pixel values indicating Bq/cm3).

Parameters that can be identified as more fundamental are energy resolution including photo peak position, coincidence timing performance, noise equivalent count rate, intrinsic spatial resolution for gamma cameras, random correction performance in PET and count rate linearity.

From a practical point of view, one could decide to only monitor image parameters in a QC programme. From a cost-benefit point of view, this could be attractive, but one runs the risk that some slow deterioration or deviations are already occurring before they are visible in the image quality.

#### Preclinical phantoms

In any QC procedure, radioactive phantoms are needed that contain a certain distribution of radioactive material such that specific performance properties of the scanner can be monitored. These can be solid sources, generally point or line sources embedded in attenuating material or phantoms containing hollow structures that can be filled with radioactive solution, generally spheres, cylinders and background compartments.

In small-animal PET, for which NEMA performance parameters exist [5], the phantoms to be used have been precisely defined. These are (i) a Na-22 point source embedded in an acrylic cube of 10-mm sides for spatial resolution and sensitivity measurements, (ii) line sources filled with a F-18 or C-11 solution, embedded in cylinders of polyethylene of different sizes (simulating mice, rats and monkeys) to measure scatter fraction, count losses and random coincidences, and (iii) an image quality phantom containing hollow cylinders of 1–5-mm diameter filled with F-18 solution, two cylindrical compartments of 8-mm diameter filled with air and non-radioactive water and a background compartment filled with F-18 solution. The image quality parameters to be determined using this phantom are (i) recovery coefficients for the differently sized rods, indicative of spatial resolution, (ii) spillover of activity into the cold air and water compartments, expressing the scatter performance, and (iii) uniformity of measured activity in the background compartment that indicates the signal-to-noise ratio.

For small-animal SPECT, several of these phantoms and corresponding measurements can be adapted from the ones used for PET, keeping in mind the already mentioned fundamental differences in single-photon detection in SPECT versus coincidence detection in PET.

It should be mentioned furthermore that spatial resolution in SPECT cannot be measured in the same, straightforward manner as in PET. In both clinical and small-animal PET, spatial resolution is measured using a point or line source, reconstructed with filtered back projection (FBP). Since FBP is a vendor-independent, straightforward reconstruction algorithm, results will be one-to-one comparable for systems of different vendors. However, for multi-pinhole SPECT systems, image reconstruction is performed using iterative algorithms that make use of the system response information including the collimator and detectors. These iterative algorithms, however, do not provide unique numbers for spatial resolution when reconstructing point or line sources. Instead, their full-width-at-half-maximum (FWHM) in iteratively reconstructed images varies strongly with the number of iterations (and subsets) being used. The general rule is that smaller FWHM is obtained for larger numbers of iterations and subsets, at the cost of increase of image noise. It is therefore recommended for small-animal SPECT to express spatial resolution in terms of recovery coefficients for small spheres or rods in image quality phantoms, instead of the FWHM of reconstructed point or line sources.

Finally, it should be mentioned that the activity present in any phantom used for calibration or for QC should match the desired accuracy related to the maximum allowable duration of the test. In general, when phantom activity has decayed to a certain extent, the phantom can still be used by allowing for a longer scan duration. One could for instance specify the minimum number of counts that should be acquired instead of the duration of the scan. Special attention, however, should be paid to the phantom activity not being comparable to the background activity in the laboratory. In that case, one could still acquire ‘enough counts’, but these are then of little value since image quality will be poor, and quantification will be inadequate.

#### Image quality phantoms

Several image quality phantoms are available for both small-animal PET and SPECT. The phantom for NEMA small-animal PET was already mentioned. Furthermore, phantoms exist containing small hollow spheres to be filled with radioactivity and inserted into a non-zero background activity compartment (Microhollow sphere phantom) and ‘Derenzo-like’ phantoms containing hot rods in a cold background or cold rods in a radioactive background (Mini Deluxe phantoms). Also, a downscaled version of the NEMA NU4 PET phantom, accommodating the high spatial resolution of multi-pinhole small-animal SPECT, has been proposed [67].

#### Discussion items

As indicated earlier, official guidelines for small-animal SPECT are still lacking, whereas those for small-animal PET could be taken from reference (Busemann Sokole, Płachcínska, & Britten, 2010; Busemann Sokole, Płachcínska, Britten, et al., 2010). In implementing QC procedures in small-animal imaging, several decisions have to be made. As a starting point, one could take the procedures in clinical practice as an example and decide which procedures should be copied to the preclinical situation. Several important issues in the clinic, such as high radiation dose to patients and wrong decisions in diagnosis or therapy, are less of an issue in preclinical research. However, generating (and publishing) scientific results that are based on sub-optimal imaging conditions or faulty processing is of course unwanted in both cases, especially in view of the current discussions about integrity and quality of scientific data.

Cost-benefit discussions in preclinical imaging are expected to be similar to those in clinical imaging. The necessity to repeat scans in case of equipment that is out of order will directly lead to additional costs for radiopharmaceuticals and personnel. In animal research, equipment that is non-usable for a too long time, can even lead to the necessity to replace the animals, also leading to additional costs and unwanted delay. As already mentioned, it is important to assess the balance between ‘image parameters’ and ‘fundamental parameters’ to be incorporated. When only focusing on image parameters, costs will probably be lower, whereas measuring all possible fundamental parameters will lead to early detection of any possible system failure but will of course increase costs.

Finally, an interesting and important discussion issue is whether our goal is to provide comprehensive guidelines for small-animal PET and SPECT imaging, or whether it is worthwhile to move a step further, and work on an accreditation programme of scanners and procedure along the lines of, for instance, the accreditation for human FDG-PET as set up by EARL (see http://earl.eanm.org).

## Conclusions

Reproducible animal experiments are very challenging due to the combination of the different factors described above. To minimize the failure of often expensive studies, it is important to attribute sufficient attention to each of the aspects described in this paper. The following is a list of items to be considered when setting up a small-animal imaging experiment:

1. Based on the main (biological) effect of interest, that needs to be investigated using an imaging experiment, select the proper imaging modality. In preclinical nuclear imaging, SPECT can provide sub-millimetre spatial resolution at sensitivities of 0.01–0.1 %, whereas PET can provide a spatial resolution of 1–2 mm but at sensitivities mostly >1 %. SPECT might have an advantage when isotopes with longer half-life are required when slower biological processes have to be longitudinally monitored. Furthermore, SPECT can offer multi-isotope imaging.
2. Based on the main (biological) effect of interest, that needs to be investigated using an imaging experiment, a correct animal model needs to be selected.
3. Select a proper control group of animals. Generally, these are healthy young adult rodents.
4. Decide on an adequate sample size based on the expected differences that should be observed between control and disease/treatment groups and the intra-animal variability of the SPECT/PET measurements.
5. Related to animal handling, a general rule is to reduce any source of variability to improve the precision of quantitative SPECT/PET measurements.
6. Thus, take attention to animal housing to avoid stress and monitor animal health.
7. If imaging requires fasting, the fasting period should be kept constant and at a minimum, ideally less than 6 h. Drinking water should not be withdrawn, and overnight fasting should be avoided.
8. During imaging experiments, animals are mostly immobilized by light anaesthesia. It is very important to select a proper anaesthesia (and dosing) because some anaesthetics may have influence on the biological effect of interest. This is especially important when investigating the brain.
9. During anaesthesia, and thus mostly during SPECT/PET/CT acquisitions, animal heating is required to maintain a constant body temperature.
10. When the tracer is administered to the animals, the volumes used for intravenous injections should not exceed specific limits. The same applies for blood volumes that have to be withdrawn in case of blood sampling.
11. After the imaging experiment, animals should be monitored during awakening from anaesthesia.
12. For accurate quantification, the acquired SPECT/PET images should be iteratively reconstructed and corrected for image degrading effects, such as attenuation, scatter and partial volume effects. Moreover, a sufficient number of iterations should be used and the reconstructed voxel size should be sufficiently small (at least smaller than the system spatial resolution).
13. When sample counting (blood/tissue) is performed, cross-calibration between dose calibrator, SPECT/PET camera and blood/gamma counter should be performed.
14. Finally, a quality control programme should be implemented to guarantee for accurate and precise SPECT and PET quantifications.

## References

[1] De Kemp RA, Epstein FH, Catana C, Tsui BMW, Ritman EL (2010). Small-animal molecular imaging methods. J Nucl Med.

[2] Del Guerra A, Belcari N (2007). State-of-the-art of PET, SPECT and CT for small animal imaging. Nucl Instrum Methods Phys Res, Sect A.

[3] Sauer UG, Spielmann H, Rusche B (2005). Fourth EU report on the statistics on the number of animals used for scientific purposes in 2002—trends, problems, conclusions. ALTEX.

[4] Deleye S, Van Holen R, Verhaeghe J, Vandenberghe S, Stroobants S, Staelens S (2013). Performance evaluation of small-animal multipinhole μSPECT scanners for mouse imaging. Eur J Nucl Med Mol Imaging.

[5] Goertzen, AL, Bao, Q, Bergeron, M, Blankemeyer, E, Blinder, S, Canadas, M, Laforest, R (2012) NEMA NU 4–2008 comparison of preclinical PET imaging systems. Journal of Nuclear Medicine. http://doi.org/10.2967/jnumed.111.099382 10.2967/jnumed.111.099382 PMC4128012 22699999

[6] Fisher M, Feuerstein G, Howells DW, Hurn PD, Kent TA, Savitz SI, Lo EH (2009). Update of the stroke therapy academic industry roundtable preclinical recommendations. Stroke.

[7] Henderson, VC, Kimmelman, J, Fergusson, D, Grimshaw, JM, & Hackam, DG (2013) Threats to validity in the design and conduct of preclinical efficacy studies: a systematic review of guidelines for in vivo animal experiments. PLoS Medicine, 10(7). http://doi.org/10.1371/journal.pmed.1001489 10.1371/journal.pmed.1001489 PMC3720257 23935460

[8] Casellas, J (2011) Inbred mouse strains and genetic stability: a review. Animal. http://doi.org/10.1017/S1751731110001667 10.1017/S1751731110001667 22440695

[9] Chia R, Achilli F, Festing MFW, Fisher EMC (2005). The origins and uses of mouse outbred stocks. Nat Genet.

[10] Hoekzema E, Herance R, Rojas S, Pareto D, Abad S, Jiménez X, Gispert JD (2010). The effects of aging on dopaminergic neurotransmission: a microPET study of [11C]-raclopride binding in the aged rodent brain. Neuroscience.

[11] Robinton, DA, & Daley, GQ (2012) The promise of induced pluripotent stem cells in research and therapy. Nature. http://doi.org/10.1038/nature10761 10.1038/nature10761 PMC3652331 22258608

[12] Gotz, J, Gotz, NN (2009) Animal models for Alzheimer’s disease and frontotemporal dementia: a perspective. ASN Neuro. http://doi.org/10.1042/AN20090042. 10.1042/AN20090042 PMC2785514 19839939

[13] Wolfer DP, Litvin O, Morf S, Nitsch RM, Lipp H-P, Würbel H (2004). Laboratory animal welfare: cage enrichment and mouse behaviour. Nature.

[14] Doulames V, Lee S, Shea TB (2013). Environmental enrichment and social interaction improve cognitive function and decrease reactive oxidative species in normal adult mice. Int J Neurosci.

[15] Martin AL, Brown RE (2010). The lonely mouse: verification of a separation-induced model of depression in female mice. Behav Brain Res.

[16] Arndt SS, Lohavech D, van’t Klooster J, Ohl F (2010). Co-species housing in mice and rats: effects on physiological and behavioral stress responsivity. Horm Behav.

[17] Pritchett-corning KR, Prins J, Feinstein R, Goodwin J, Nicklas W, Riley L (2014). AALAS/FELASA working group on health monitoring of rodents for animal transfer. J Am Assoc Lab Anim Sci.

[18] Hawkins P, Morton DB, Burman O, Dennison N, Honess P, Jennings M, Westwood K (2011). A guide to defining and implementing protocols for the welfare assessment of laboratory animals: eleventh report of the BVAAWF/FRAME/RSPCA/UFAW Joint Working Group on Refinement. Lab Anim.

[19] Alf MF, Duarte JMN, Schibli R, Gruetter R, Krämer SD (2013). Brain glucose transport and phosphorylation under acute insulin-induced hypoglycemia in mice: an 18 F-FDG PET study. J Nucl Med.

[20] Wong K-P, Sha W, Zhang X, Huang S-C (2011). Effects of administration route, dietary condition, and blood glucose level on kinetics and uptake of 18 F-FDG in mice. J Nucl Med.

[21] Jensen TL, Kiersgaard MK, Sørensen DB, Mikkelsen LF (2013). Fasting of mice: a review. Lab Anim.

[22] Mahling, M, et al. (2015) A comparative pO2 probe and [18F]-fluoro-azomycinarabino-furanoside ([18F]FAZA) PET study reveals anesthesia-induced impairment of oxygenation and perfusion in tumor and muscle. PLoS One. http://doi.org/10.1371/journal.pone.0124665 10.1371/journal.pone.0124665 PMC4406741 25902054

[23] Cesarovic N (2010). Isoflurane and sevoflurane provide equally effective anaesthesia in laboratory mice. Lab Anim.

[24] Field KJ, White WJ, Lang CM (1993). Anaesthetic effects of chloral hydrate, pentobarbitone and urethane in adult male rats. Lab Anim.

[25] Hara K, Harris RA (2002). The anesthetic mechanism of urethane: the effects on neurotransmitter-gated ion channels. Anesth Analg.

[26] Hildebrandt IJ, Su H, Weber WA (2008). Anesthesia and other considerations for in vivo imaging of small animals. Ilar J.

[27] Alstrup AKO, Smith DF (2013). Anaesthesia for positron emission tomography scanning of animal brains. Lab Anim.

[28] Elfving B, Bjørnholm B, Knudsen GM (2003). Interference of anaesthetics with radioligand binding in neuroreceptor studies. Eur J Nucl Med Mol Imaging.

[29] Momosaki S, Hatano K, Kawasumi Y, Kato T, Hosoi R, Kobayashi K, Ito K (2004). Rat-PET study without anesthesia: anesthetics modify the dopamine D 1 receptor binding in rat brain. Synapse.

[30] Diehl KH (2001). A good practice guide to the administration of substances and removal of blood, including routes and volumes. J Appl Toxicol.

[31] Turner PV, Pekow C, Vasbinder MA, Brabb T (2011). Administration of substances to laboratory animals: equipment considerations, vehicle selection, and solute preparation. J Am Assoc Lab Anim Sci.

[32] Li P, Zhao L (2007). Developing early formulations: practice and perspective. Int J Pharm.

[33] Golde WT, Gollobin P, Rodriguez LL (2005). A rapid, simple, and humane method for submandibular bleeding of mice using a lancet. Lab Anim.

[34] Heimann M, Käsermann HP, Pfister R, Roth DR, Bürki K (2009). Blood collection from the sublingual vein in mice and hamsters: a suitable alternative to retrobulbar technique that provides large volumes and minimizes tissue damage. Lab Anim.

[35] Alf MF, Wyss MT, Buck A, Weber B, Schibli R, Krämer SD (2013). Quantification of brain glucose metabolism by 18 F-FDG PET with real-time arterial and image-derived input function in mice. J Nucl Med.

[36] Aide N, Visser EP, Lheureux S, Heutte N, Szanda I, Hicks RJ (2012). The motivations and methodology for high-throughput PET imaging of small animals in cancer research. Eur J Nucl Med Mol Imaging.

[37] Eckelman WC (2008). Further discussions on choosing the number of animals for an experiment. Nucl Med Biol.

[38] Eckelman WC, Kilbourn MR, Joyal JL, Labiris R, Valliant JF (2007). Justifying the number of animals for each experiment. Nucl Med Biol.

[39] Dandekar M, Tseng JR, Gambhir SS (2007). Reproducibility of 18 F-FDG microPET studies in mouse tumor xenografts. J Nucl Med.

[40] Cherry SR (2004). In vivo molecular and genomic imaging: new challenges for imaging physics. Phys Med Biol.

[41] Franc BL, Acton PD, Mari C, Hasegawa BH (2008). Small-animal SPECT and SPECT/CT: important tools for preclinical investigation. J Nucl Med.

[42] Meikle SR, Kench P, Kassiou M, Banati RB (2005). Small animal SPECT and its place in the matrix of molecular imaging technologies. Phys Med Biol.

[43] Phelps ME (2000). Positron emission tomography provides molecular imaging of biological processes. Proc Natl Acad Sci U S A.

[44] Yao, R, Lecomte, R, & Crawford, ES (2012) Small-animal PET: what is it, and why do we need it? Journal of Nuclear Medicine Technology. http://doi.org/10.2967/jnmt.111.098632 10.2967/jnmt.111.098632 22582006

[45] Rogulski, MM, Barber, HB, Barrett, HH, Shoemaker, RL, & Woolfenden, JM (1993) Ultra-high-resolution brain SPECT imaging: simulation results. IEEE Transactions on Nuclear Science, 40(4). http://doi.org/10.1109/23.256722

[46] Schramm, NU, Ebel, G, Engeland, U, Schurrat, T, Behe, M., & Behr, TM (2002) High-resolution SPECT using multi-pinhole collimation. IEEE Nuclear Science Symposium Conference Record, 2. http://doi.org/10.1109/NSSMIC.2002.1239437

[47] Van Audenhaege K, Vanhove C, Vandenberghe S, Van Holen R (2015). The evaluation of data completeness and image quality in multiplexing multi-pinhole SPECT. IEEE Trans Med Imaging.

[48] Boisson F, Zahra D, Parmar A, Gregoire M-C, Meikle SR, Hamse H, Reilhac A (2013). Imaging capabilities of the Inveon SPECT system using single-and multipinhole collimators. J Nucl Med.

[49] Matsunari I, Miyazaki Y, Kobayashi M, Nishi K, Mizutani A, Kawai K, Kinuya S (2014). Performance evaluation of the eXplore speCZT preclinical imaging system. Ann Nucl Med.

[50] Van der Have F, Vastenhouw B, Ramakers RM, Branderhorst W, Krah JO, Ji C, Beekman FJ (2009). U-SPECT-II: an ultra-high-resolution device for molecular small-animal imaging. J Nucl Med.

[51] Stickel JR, Qi J, Cherry SR (2007). Fabrication and characterization of a 0.5-mm lutetium oxyorthosilicate detector array for high-resolution PET applications. J Nucl Med.

[52] Rafecas M, Böning G, Pichler BJ, Lorenz E, Schwaiger M, Ziegler SI (2001). A Monte Carlo study of high-resolution PET with granulated dual-layer detectors. IEEE Transactions on Nuclear Science.

[53] Lange K, Carson R (1984). EM reconstruction algorithms for emission and transmission tomography. J Comput Assist Tomogr.

[54] España S, Marcinkowski R, Keereman V, Vandenberghe S, Van Holen R (2014). DigiPET: sub-millimeter spatial resolution small-animal PET imaging using thin monolithic scintillators. Phys Med Biol.

[55] Herrmann K, Dahlbom M, Nathanson D, Wei L, Radu C, Chatziioannou A, Czernin J (2013). Evaluation of the Genisys4, a bench-top preclinical PET scanner. J Nucl Med.

[56] Frey EC, Humm JL, Ljungberg M (2012). Accuracy and precision of radioactivity quantification in nuclear medicine images. Semin Nucl Med.

[57] Vandeghinste, B., Van Holen, R., Vanhove, C., De Vos, F., Vandenberghe, S., & Staelens, S. (2014) Use of a ray-based reconstruction algorithm to accurately quantify preclinical microspect images. Molecular Imaging, 13(4). 24824961

[58] Ogawa K, Harata Y, Ichihara T, Kubo A, Hashimoto S (1991). A practical method for position-dependent Compton-scatter correction in single photon emission CT. IEEE Trans Med Imaging.

[59] Prasad R, Zaidi H (2014). Scatter characterization and correction for simultaneous multiple small-animal PET imaging. Mol Imaging Biol.

[60] Vanhove C, Defrise M, Bossuyt A, Lahoutte T (2011). Improved quantification in multiple-pinhole SPECT by anatomy-based reconstruction using microCT information. Eur J Nucl Med Mol Imaging.

[61] Feldkamp, LA, Davis, LC, & Kress, JW (1984) Practical cone-beam algorithm. Journal of the Optical Society of America A. http://doi.org/10.1364/JOSAA.1.000612

[62] Flannery BP, Deckman HW, Roberge WG, D’Amico KL (1987). Three-dimensional X-ray microtomography. Science (New York, NY).

[63] Sasov A (2001). Micro-CT for nondestructive 3D reconstruction of MEMS and sensors. Mems Design, Fabrication, Characterization, and Packaging.

[64] Boellaart R (2010). FDG PET and PET/CT: EANM procedure guidelines for tumour PET imaging: version 1.0. Eur J Nucl Med Mol Imaging.

[65] Busemann Sokole E, Płachcínska A, Britten A (2010). Acceptance testing for nuclear medicine instrumentation. Eur J Nucl Med Mol Imaging.

[66] Busemann Sokole E, Płachcínska A, Britten A, Lyra Georgosopoulou M, Tindale W, Klett R (2010). Routine quality control recommendations for nuclear medicine instrumentation. Eur J Nucl Med Mol Imaging.

[67] Visser EP, Harteveld A a, Meeuwis APW, Disselhorst J a, Beekman FJ, Oyen WJG, Boerman OC (2011). Image quality phantom and parameters for high spatial resolution small-animal SPECT. Nucl Instrum Methods Phys Res, Sect A.

